# Using a Web-Based Application to Define the Accuracy of Diagnostic Tests When the Gold Standard Is Imperfect 

**DOI:** 10.1371/journal.pone.0079489

**Published:** 2013-11-12

**Authors:** Cherry Lim, Prapass Wannapinij, Lisa White, Nicholas P. J. Day, Ben S. Cooper, Sharon J. Peacock, Direk Limmathurotsakul

**Affiliations:** 1 Mahidol-Oxford Tropical Medicine Research Unit, Faculty of Tropical Medicine, Mahidol University, Bangkok, Thailand; 2 Centre for Clinical Vaccinology and Tropical Medicine, Nuffield Department of Clinical Medicine, University of Oxford, Oxford, United Kingdom; 3 Department of Medicine, University of Cambridge, Cambridge, United Kingdom; 4 Department of Microbiology and Immunology, Faculty of Tropical Medicine, Mahidol University, Bangkok, Thailand; 5 Department of Tropical Hygiene, Faculty of Tropical Medicine, Mahidol University, Bangkok, Thailand; National Taiwan University, Taiwan

## Abstract

**Background:**

Estimates of the sensitivity and specificity for new diagnostic tests based on evaluation against a known gold standard are imprecise when the accuracy of the gold standard is imperfect. Bayesian latent class models (LCMs) can be helpful under these circumstances, but the necessary analysis requires expertise in computational programming. Here, we describe open-access web-based applications that allow non-experts to apply Bayesian LCMs to their own data sets via a user-friendly interface.

**Methods/Principal Findings:**

Applications for Bayesian LCMs were constructed on a web server using R and WinBUGS programs. The models provided (http://mice.tropmedres.ac) include two Bayesian LCMs: the two-tests in two-population model (Hui and Walter model) and the three-tests in one-population model (Walter and Irwig model). Both models are available with simplified and advanced interfaces. In the former, all settings for Bayesian statistics are fixed as defaults. Users input their data set into a table provided on the webpage. Disease prevalence and accuracy of diagnostic tests are then estimated using the Bayesian LCM, and provided on the web page within a few minutes. With the advanced interfaces, experienced researchers can modify all settings in the models as needed. These settings include correlation among diagnostic test results and prior distributions for all unknown parameters. The web pages provide worked examples with both models using the original data sets presented by Hui and Walter in 1980, and by Walter and Irwig in 1988. We also illustrate the utility of the advanced interface using the Walter and Irwig model on a data set from a recent melioidosis study. The results obtained from the web-based applications were comparable to those published previously.

**Conclusions:**

The newly developed web-based applications are open-access and provide an important new resource for researchers worldwide to evaluate new diagnostic tests.

## Introduction

The accuracy (i.e. sensitivity and specificity) of new diagnostic tests are usually defined against an established gold standard. This assumes that the gold standard is perfect (100% sensitive and specific), but this is not always the case. Gold standard tests for many diseases are of unknown accuracy, may be too invasive, or may not be available [1,2,3]. For example, expert microscopy is used as the gold standard during the evaluation of alternative tests for malaria, but the accuracy of an individual microscopist is usually unknown and could be imperfect [4,5]. A pathological diagnosis made from tissue is a gold standard for cancer diagnosis, but access to tissue is invasive and only obtained when the suspicion for cancer is high, which is problematic for the evaluation of alternative diagnostic tests [6,7]. There is no gold standard for the diagnosis of latent tuberculosis infection (LTBI), and the accuracy of available diagnostic tests for this remain uncertain [8,9]. 

If the error rates of a gold standard are ignored during the evaluation of new diagnostic tests, the accuracy of new tests can be underestimated and disease prevalence either under- or over- estimated [10,11]. The impact of an imperfect gold standard can be demonstrated using a hypothetical example in which 200 subjects with a true disease prevalence of 50% (100 diseased subjects and 100 non-diseased subjects) are evaluated. If the true sensitivity and specificity of the current gold standard are 80% and 100%, respectively, the estimated prevalence of the disease using the gold standard will be 40% (80/200) rather than 50%. If the true sensitivity and specificity of a newly developed diagnostic test are 95% and 100%, respectively, these will be incorrectly estimated against this imperfect gold standard as 95% (76/80) and 84% (101/120), respectively, and the test may be erroneously discarded. 

In 1980, Hui and Walter proposed the first statistical model to estimate the accuracy of diagnostic tests when the accuracy of the gold standard is unknown [12]. Their model does not assume that the gold standard is perfect, but calculates the accuracy of diagnostic tests based on the estimation of true disease prevalence. Their approach requires that two diagnostic tests are both applied to two populations with differing disease prevalence. The result of one diagnostic test is assumed to have no effect on that of the other, and the accuracy of both diagnostic tests is assumed to be consistent among two different populations [12]. Disease prevalence in both populations and the accuracy of both diagnostic tests can then be estimated using the formula provided [12]. Based on the same principle, in 1988 Walter and Irwig expanded the model for the application of three diagnostic tests in one population [13]. 

In 1995, Joseph et al. proposed the use of Bayesian latent class models (LCMs) as a method to estimate the accuracy of diagnostic tests when the accuracy of the gold standard is unknown [14]. Bayesian LCMs are applicable to both the Hui and Walter model and the Walter and Irwig model [3,15]. Bayesian LCMs have been increasingly used to evaluate the accuracy of diagnostic tests for both communicable diseases (e.g. malaria [5,16,17], tuberculosis [18] and cholera [19]) and non-communicable diseases (e.g. breast cancer [7], temporal arteritis [20] and neurocognitive disorders [21]). We recently showed that gold standard tests for melioidosis (culture) [22], leptospirosis (a combination of culture and MAT) [23] and dengue infection (paired ELISAs) [24] have low sensitivities, and that Bayesian LCMs are useful for estimating the true accuracy of alternative diagnostic tests when the accuracy of the gold standard is unknown. An important drawback is that computation of Bayesian LCM requires considerable expertise and specific mathematical software such as R and WinBUGS [25,26]. Commonly used statistical software such as SAS, SPSS, EpiInfo^TM^ and STATA do not contain the commands for Bayesian LCMs. These requirements may deter researchers from using Bayesian LCMs. At the present time, there is only one application which allows users to apply Bayesian LCMs to their own data sets without the need for specialist mathematical software [27]. However, this application requires users to download and install another three programs including WinBUGS, Active Perl and Microsoft.Net Framework. In addition, its interface can be difficult for first-time users with a limited mathematical or statistical background [27]. 

Here, we describe the development of user-friendly, open-access, web-based applications that can compute imperfect gold standard models using Bayesian LCMs. We provide both simplified and advanced interfaces so that the novice can use these readily and advanced users can adjust settings as required. 

## Results and Discussion

### Web-based application

**Figure 1:**
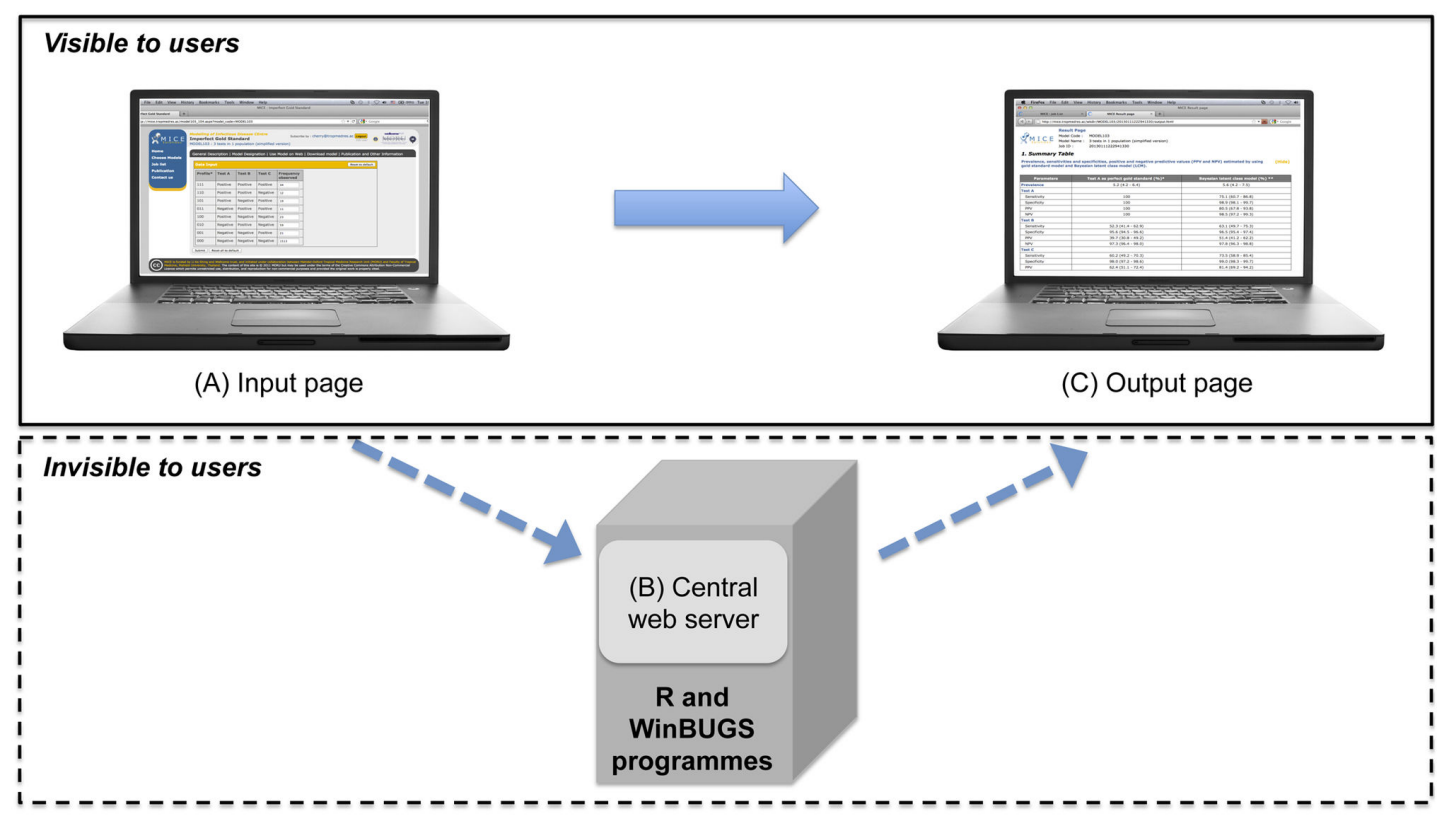
Schematic diagram of the web-based application (http://mice.tropmedres.ac). (A) Users input the data set and settings into a table provided on the webpage, (B) The central web server invisibly transforms the data set and settings inputted into multiple text files suitable for the statistical software, and automatically runs the Bayesian latent class models (LCM) using the R and WinBUGS programs. (C) The results estimated by Bayesian LCM are provided on the webpage within few minutes.

A schematic diagram of the web application and the programs running on the central server is shown in Figure 1. The web-based application consists of two major components. The first is a webpage (http://mice.tropmedres.ac) that receives data inputs in a simple tabular format for Bayesian LCMs (two-tests in the two-population model (Hui and Walter model) [12], and three-tests in the one-population model (Walter and Irwig model) [13]). The second is an application on our central web server that invisibly converts data inputs into text files that are suitable for mathematical programs, and automatically performs Bayesian LCMs using R and WinBUGS programmes. The user receives their results on the webpage within a few minutes. Our web-based applications do not require users to download or install any software. 

### Bayesian Latent Class model (LCM)

 Data sets are applicable to Bayesian LCMs if: (1) two diagnostic tests are applied together to more than one population; (2) more than two diagnostic tests are applied together to one population; or (3) more than two diagnostic tests are applied together to more than one population [14,28]. This is because Bayesian LCMs need to estimate true disease prevalence, and a 2x2 summary table of two diagnostic tests applied to one population does not provide enough data for this calculation [14,28]. In the event that two diagnostic tests were applied together to one population, it is possible to divide a single population data set into multiple population data sets based on specific variables [29]. For example, a data set of one population may be divided into multiple populations based on different geographical regions in the event that spatial data has been collected [29]. Selecting diagnostic tests to include in the Bayesian LCM model is very important, and the aim should be to include tests that diagnose the same disease based on different biological assays [28,29]. For example, antigen detection, antibody detection and imaging of a disease could be considered as different biological assays of a single disease. 

Figure S1 illustrates how the Bayesian LCM estimates actual accuracies of diagnostic tests. In brief, these do not assume that any test or a combination of any tests is perfect, but considers that each test could be imperfect in diagnosing the true disease status. The true disease status of the patient population is then defined on the basis of overall prevalence. The model estimates the prevalence and accuracy of each test based on the observed frequency of the possible combinations of test result [14,28,30]. The model is then iterated using the Markov chain Monte Carlo (MCMC) method to estimate all unknown parameters, including prevalence and accuracy of each diagnostic test, and their 95% credible intervals [31]. 

### Simplified web-based interfaces including practice data sets

**Figure 2:**
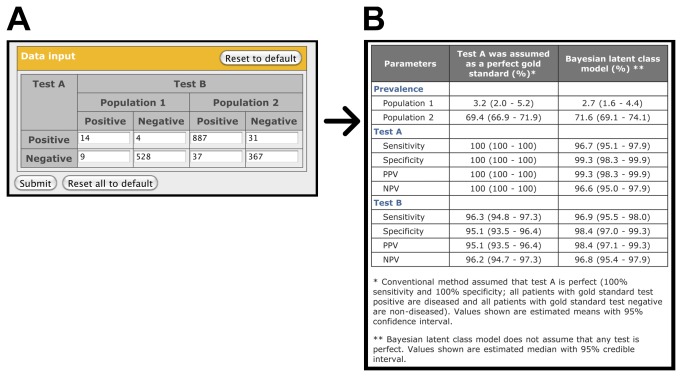
Input and output screen for the simplified interface of the two-tests in two-population model (Hui and Walter model) provided on the website (http://mice.tropmedres.ac). See text for details.

Simplified interfaces have been created in which all settings of the Bayesian LCMs are set in default mode and hidden from view. Practice data sets are provided to allow the user to gain experience in use of the website prior to analysing their own data set. For the two-tests in the two-population model, the practice data set is an application of the Mantoux (test A) and Tine (test B) tests to diagnose tuberculosis in 555 participants in a southern U.S. school district (population 1) and 1322 participants at the Missouri State Sanatorium (population 2) [12]. The input data set consists of 8 numbers in a tabular format describing the summary results (Figure 2A). The output page (Figure 2B) shows that the prevalence of tuberculosis in the two populations estimated by the Bayesian LCM (2.8% and 71.6%) were different from those based on test A alone (3.2% and 69.4%, respectively). In addition, the Bayesian LCM estimated that the true specificity of test B was 98.3%, which is higher than 95.1% estimated for test A. The specificity of test B was underestimated when compared with test A because the true sensitivity of test A was less than perfect (96.6%). The imperfect sensitivity of test A was validated using another data set of patients with culture-positive pulmonary tuberculosis [12]. The results obtained by our web-based application using the Bayesian LCM were very similar to those calculated by the formulas described by Hui and Walter [12] (Table S1).

**Figure 3:**
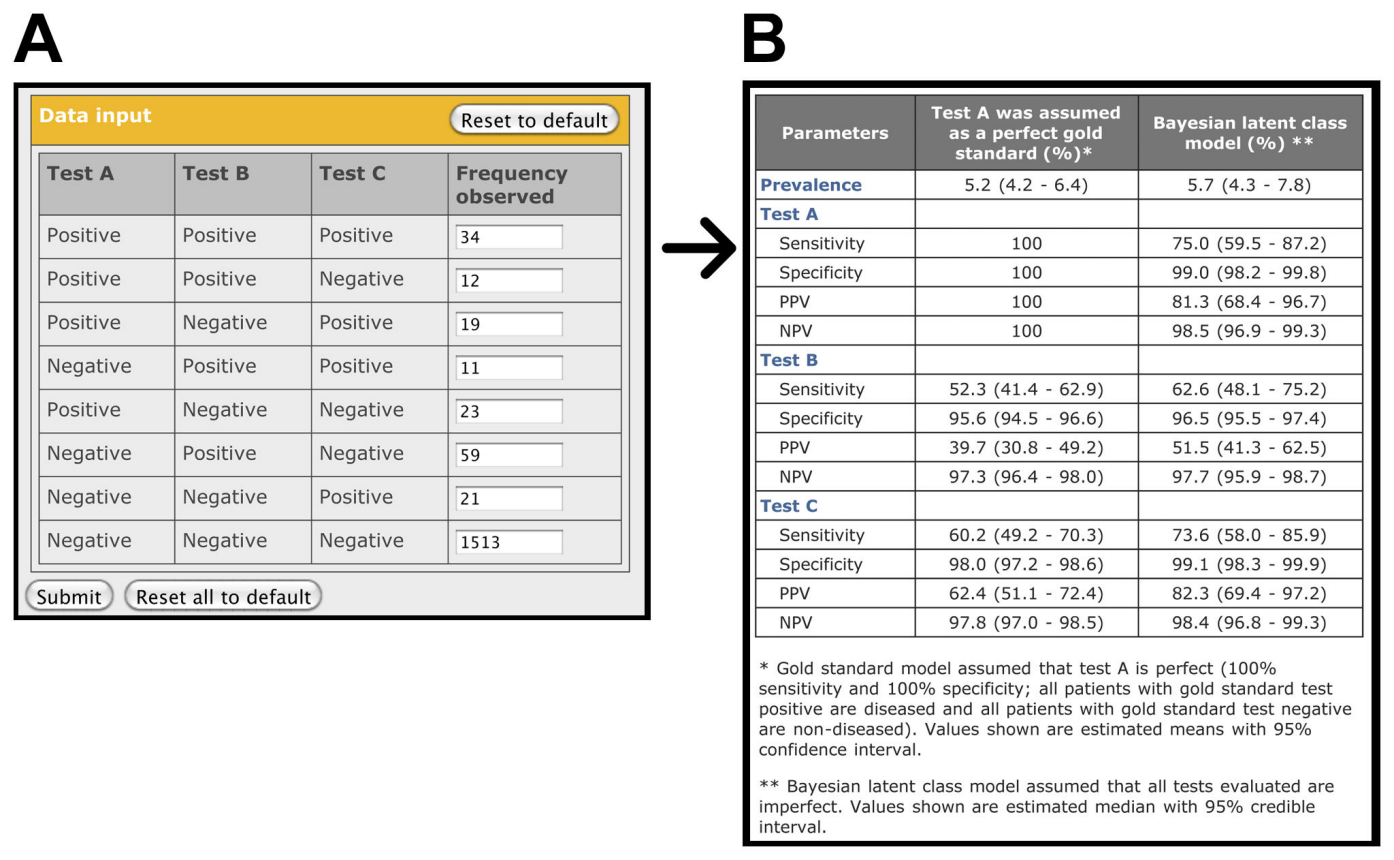
Input and output screen for the simplified interface of the three-tests in one-population model (Walter and Irwig model) provided on the website (http://mice.tropmedres.ac). See text for details.

For the three-tests in one-population model, the practice data set is an assessment of pleural thickening by three independent radiologists (test A, B and C) for 1,692 male employees in asbestos mines and mills (one population) [13]. The input data set consists of 8 numbers in a tabular format describing the summary results (Figure 3A). The output page (Figure 3B) shows that the true sensitivity of radiologist B (63.1%) and radiologist C (73.5%) were much higher than those estimated by considering radiologist A to be the gold standard (52.3% and 60.2%, respectively). This is because the true sensitivity of radiologist A was estimated to be only 75.1%. The results obtained by our web-based application using Bayesian LCM were very similar to those calculated by the maximum likelihood estimation methods described by Walter and Irwig [13] (Table S2).

### Advanced web-based interfaces

Advanced interfaces for both models were designed for those with experience in Bayesian statistics who wish to adjust the default settings of the models. Adjustable settings of both models include: (1) an additional assumption that there is a correlation among the diagnostic tests being evaluated; (2) adding a priori scientific knowledge about prevalence and accuracy of diagnostic tests into the analysis (i.e., adjusting prior distributions and probable ranges of all unknown parameters); (3) defining starting values of prevalence and accuracy of diagnostic tests for the first iteration of the MCMC method (i.e., defining initial values of all unknown parameters); (4) defining the total number of iterations at the beginning of an MCMC run to be discarded (i.e., burn-in iterations) and the total number of iterations to be used for estimating values of the unknown parameters (Table S3 and S4). 

The ability to define a correlation among diagnostic tests in the model could be useful because ignoring this can lead to inaccurate estimation of test accuracy [14,28]. This is of particular concern when the diagnostic tests being evaluated are based on a comparable biological assay. For example, culture and PCR are based on organism detection despite different methods. The correlation between culture and PCR (if present) means that diseased patients who are positive for culture are likely to be positive for PCR, an assumption with biological plausibility. In addition, the ability to take account of external information on beliefs about test accuracy before the data set is analysed (specified in the prior distribution) is a key part of Bayesian statistics [32]. Beliefs relating to parameters are usually presented as probability distributions, and a beta distribution is used here to represent the probability distributions of prevalence, test sensitivity and test specificity [32]. The beta distribution is characterized by two positive numbers, such as beta distribution (1,1) or beta distribution (90,10), to express the shape of its probability distribution within a range between 0 and 1. The probability distribution can also be truncated on the interval defined. The default setting for the simplified interface assumes that we know nothing about diagnostic tests before the data set is analysed; in other words, non-informative prior distribution is used for all parameters (beta distribution (0.5,0.5)), except a certainty that specificity is above 0.4 (permitted ranges of specificities are between 0.4 and 1). Beta distribution (0.5, 0.5) implies that every value of the unknown parameter is equally likely prior to the analysis. Truncation of probable ranges of specificities prevents the Bayesian LCM from estimating the test accuracy the other way around (considering a test with true sensitivity of 95% and specificity of 95% as a test with sensitivity of 5% and specificity of 5%) [12], and relies on an assumption that users are not using tests with very low specificities (tests with high false positive rate in healthy individuals) in their studies. In the advanced interface, the user can define the two positive numbers for each beta distribution prior and a probable range of each parameter estimated. This is recommended when external information or beliefs about test accuracy are available and reliable, because that information (informative priors) can improve the accuracy and precision of all parameters estimated in Bayesian LCMs [3,32,33]. For example, culture positivity for pathogenic organisms from blood specimens that are rarely isolated as contaminants could be considered highly specific for many bacterial and fungal infections. Therefore, the specificity of culture could be fixed at 100% in previous studies [22,23], and this can be taken into account via prior distributions as shown in the following example. 

### Examples of advanced interfaces

The utility of the advanced interfaces is illustrated here using the data set from a recent melioidosis study performed by us [22] (Figure S2). In brief, the study prospectively recruited patients with suspected melioidosis presenting at the Sappasithiprasong Hospital, Ubon Ratchathani, Northeast Thailand between June and October 2004 [22]. A total of 320 patients were included in the study, and blood specimens were collected on admission and evaluated for 5 diagnostic tests (bacterial culture, indirect hemagglutination test (IHA), IgM immunochromogenic cassette test (ICT), IgG ICT, and the ELISA). Isolation of *B. pseudomallei* from any clinical specimen (including blood, urine, sputum and pus) was defined as bacterial culture positive [34]. IHA, IgM ICT, IgG ICT and ELISA were serological tests [35,36]. 

The advanced interface was applied to multiple example data sets generated from the complete data set of five diagnostic tests [22]. As the three-tests in one-population model was used, we initially created all possible permutations of three-tests from the five-tests data set. In addition, as diagnostic tests with different diagnostic biological phenomena should be included in the model, combinations of culture and two serological tests were selected. This made 6 example data sets, including (1) Culture, IHA and IgM ICT (2), Culture, IHA and IgG ICT (3), Culture, IHA and ELISA (4), Culture, IgM ICT and IgG ICT (5), Culture, IgM ICT and ELISA, and (6) Culture, IgG ICT and ELISA (Text S1). The setting of the model was modified from the default as follows: the specificity of culture was fixed at 100%, and there was a correlation between the two serological tests in diseased patients (Figure S2). This setting was based on biological plausibility and validated as previously described [22]. 

Table 1 shows the prevalence and accuracy of each diagnostic test estimated by the Bayesian LCM compared to those based on gold standard (culture). Results from all 6 example data sets estimated by the Bayesian LCM differed considerably from those based on the gold standard. The prevalence of melioidosis was estimated to be about 59.9% (ranging from 52.6% to 63.8%, estimated by the example data set 5 and 4, respectively), much higher than the estimated 37.2% based on culture. All six examples estimated that sensitivity of culture was only about 62.2% (ranging from 58.2% to 70.5% estimated by the example data set 4 and 5, respectively). The high prevalence and low sensitivity of culture were credible and validated by post-hoc model validation as previously described [22]. A very low specificity of ELISA (73.1%) was previously reported when compared to culture, and it had been erroneously discarded [36]. However, all example data sets that included ELISA in the model (data set 3, 5 and 6) showed that the true specificity of ELISA was about 95.2% (ranged from 90.6% to 98.3%, estimated by the example data set 5 and 6, respectively), representing a test that could be used to rule in melioidosis with a high degree accuracy. The differences among the results obtained using the 6 example data sets were minimal. All showed that culture was an imperfect gold standard, and that the accuracy of alternative diagnostic tests should be estimated by imperfect gold standard models. The results of all 6 example data sets obtained by our web-based applications were very similar to those obtained by the full data set previously described [22]. This example also shows that different combinations of diagnostic tests should provide comparable outcomes if the diagnostic tests included in the models are selected based on reasonable scientific background. 

**Table 1:** Prevalence, sensitivities and specificities estimated by using culture as a perfect gold standard and the complete data set, and by Bayesian latent class models using advance interfaces of the web-based applications (www.mice.tropmedres.ac) and 6 example data sets extracted from the complete data set.

Parameters	Culture as gold	Example	Example	Example	Example	Example	Example
	standard ^a^	Data set 1 ^b^	Data set 2 ^b^	Data set 3 ^b^	Data set 4 ^b^	Data set 5 ^b^	Data set 6 ^b^
Prevalence	37.2 (31.9-42.7)	62.6 (53.1-72.6)	63.1 (55.4-71.6)	55.4 (48.4-62.2)	63.8 (55.2-72.6)	52.6 (44.1-60.8)	57.2 (51.0-63.7)
Culture							
Sensitivity	100	59.4 (49.4-70.0)	58.8 (49.9-67.5)	67.1 (58.4-75.7)	58.2 (49.2-67.6)	70.5 (60.2-82.2)	64.9 (56.9-72.3)
Specificity	100	100	100	100	100	100	100
IHA							
Sensitivity	71.4 (63.2-79.7)	70.3 (61.6-78.1)	70.7 (62.0-78.3)	71.3 (63.4-78.5)	NA	NA	NA
Specificity	63.7 (57.0-70.4)	86.0 (75.0-82.5)	87.1 (78.5-93.9)	78.0 (70.0-85.2)	NA	NA	NA
IgM ICT							
Sensitivity	81.5 (74.4-88.6)	81.0 (73.4-87.4)	NA	NA	80.5 (72.9-87.0)	80.8 (73.4-87.2)	NA
Specificity	48.8 (41.8-55.7)	68.4 (56.7-82.5)	NA	NA	69.6 (58.7-80.3)	58.2 (49.3-67.0)	NA
IgG ICT							
Sensitivity	87.4 (81.3-93.4)	NA	86.7 (79.9-93.9)	NA	86.8 (79.8-92.1)	NA	87.4 (80.9-92.5)
Specificity	49.3 (42.3-56.2)	NA	74.1 (62.6-87.4)	NA	75.2 (62.7-89.4)	NA	66.6 (57.7-74.8)
ELISA							
Sensitivity	82.5 (75.4-89.3)	NA	NA	81.9 (74.4-88.0)	NA	81.7 (73.9-88.0)	81.6 (74.0-87.9)
Specificity	73.1 (67.0-79.3)	NA	NA	95.2 (87.3-99.7)	NA	90.6 (80.3-98.9)	98.3 (93.5-99.9)

Values shown are median estimates with 95% credible interval unless otherwise stated. NA = Not available.

^a^ Values shown are mean estimates with 95% confidence interval.

^b^ Each data set had a total sample size of 320 patients with three diagnostic test results (Text S1). Advanced interface of the three-tests in one-population model (Walter and Irwig model) was used, in which specificity of culture was fixed at 100%, and there was a correlation between the serological tests in diseased patient.

### Potential issues

 Before using the result estimated by the Bayesian LCM, Bayesian statistics requires that users check for convergence of the Markov chains and fitness of the model used [37]. Simple figures and guidelines on how to check for these points are always provided for users together with the results. The result shown in the summary table should not be used if the Markov chains do not converge. 

Bayesian LCMs do not assume that the accuracy of gold standard is perfect, but some assumptions are still needed. These are that each participant is assumed to contribute exactly one record (i.e. no repeated records), each participant is assumed to have been randomly selected from the population being evaluated, and the accuracy of diagnostic tests is estimated based on the overall prevalence of the disease in the study population. For the Hui and Walter model, it is also assumed that the accuracy of diagnostic tests is consistent between two populations with a different prevalence of the disease. However, it is not uncommon that the accuracy of diagnostic tests might change according to the prevalence and range of disease manifestations, and the summary statistics obtained would then be a compromise between its accuracy in the two different populations [29]. In addition, if the difference in prevalence of disease in the two populations is small, the accuracy and precision of the estimates obtained by Hui and Walter model could be very poor [29]. 

Bayesian LCM is only one of the methods recommended when the accuracy of the gold standard is imperfect or unknown [2,38]. Other methods, such as assessment of the ability of a test to predict patient outcome or assessment of the concordance of difference tests instead of test accuracy should also be considered [2,38]. In addition, accuracy of parameters estimated using Bayesian LCMs should be considered carefully and validated with all external knowledge and scientific information available [22,23]. For example, three diagnostic tests for LTBI could be applied to a large group of LTBI suspected patients, and then the three-tests in one-population model (Figure 3) can be used. If possible, any treatment provided should be the same regimen to all study patients. Then, the accuracy of diagnostic tests estimated using Bayesian LCM could be compared and validated with further evidence such as long-term outcome of the study patients who have different test results. This concept could be implemented in large cohorts or clinical trials of LTBI suspected patients.

### Further developments

 We aim to include four-tests in one-population model and five-tests in one-population model, and to include correlations among three or four diagnostic tests in those developing models. This would allow advanced users to apply Bayesian LCM with more complicated data sets in the future [22,23,24]. 

## Materials and Methods

 The web application is located at http://mice.tropmedres.ac. The interface was developed using Microsoft Visual Studio 2008 and ASP.NET 3.5 (Microsoft; Washington, US). The Bayesian statistics were processed using R version 2.11.1, RtoWinBUGS application version 2.1.16, and WinBUGS version 1.4.3 (Cambridge UK) [25,26]. All data were stored in Microsoft SQL Server 2008 R2. The applications were tested with multiple data sets including the Hui and Walter data set [12], Walter and Irwig data set [13] and melioidosis data sets [22]. Web pages were tested with Internet Explorer 9.0, Firefox 6.0.2 and Safari 5.0.2. 

## Supporting Information

Figure S1 **Schematic illustration of the use of Bayesian latent class model (LCM) to obtain unbiased estimates of accuracy of diagnostic tests.**
(TIF)

Figure S2 **Input screen for the advanced interface of three-tests in one-population model (Walter and Irwig model) provided on the website (http://mice.tropmedres.ac).**
(TIF)

Table S1 **Prevalence, sensitivities and specificities for an example data set estimated by the Bayesian latent class model (LCM) using web-based applications and by the formula originally described by Hui and Walter (two-tests in two-population model).**
(DOCX)

Table S2 **Prevalence, sensitivities and specificities for an example data set estimated by the Bayesian latent class model (LCM) using web-based applications and by the maximum likelihood method described by Walter and Irwig (three-tests in one-population model).**
(DOCX)

Table S3 **Settings for the Bayesian latent class models (LCMs) used in the web-based application (http://mice.tropmedres.ac) for two-tests in two-population model (Hui and Walter model).**
(DOCX)

Table S4 **Settings for the Bayesian latent class models (LCMs) used in web-based application (http://mice.tropmedres.ac) for three-tests in one-population model (Walter and Irwig model).**
(DOCX)

Text S1 **Six example data sets of melioidosis suspected patients.**
(DOCX)
