# Supplementary figures and images for: Using a Web-Based Application to Define the Accuracy of Diagnostic Tests When the Gold Standard Is Imperfect
Source: PLoS One. 2013 Nov 12;8(11):e79489. doi: 10.1371/journal.pone.0079489 (PMC3827152; doi:10.1371/journal.pone.0079489)

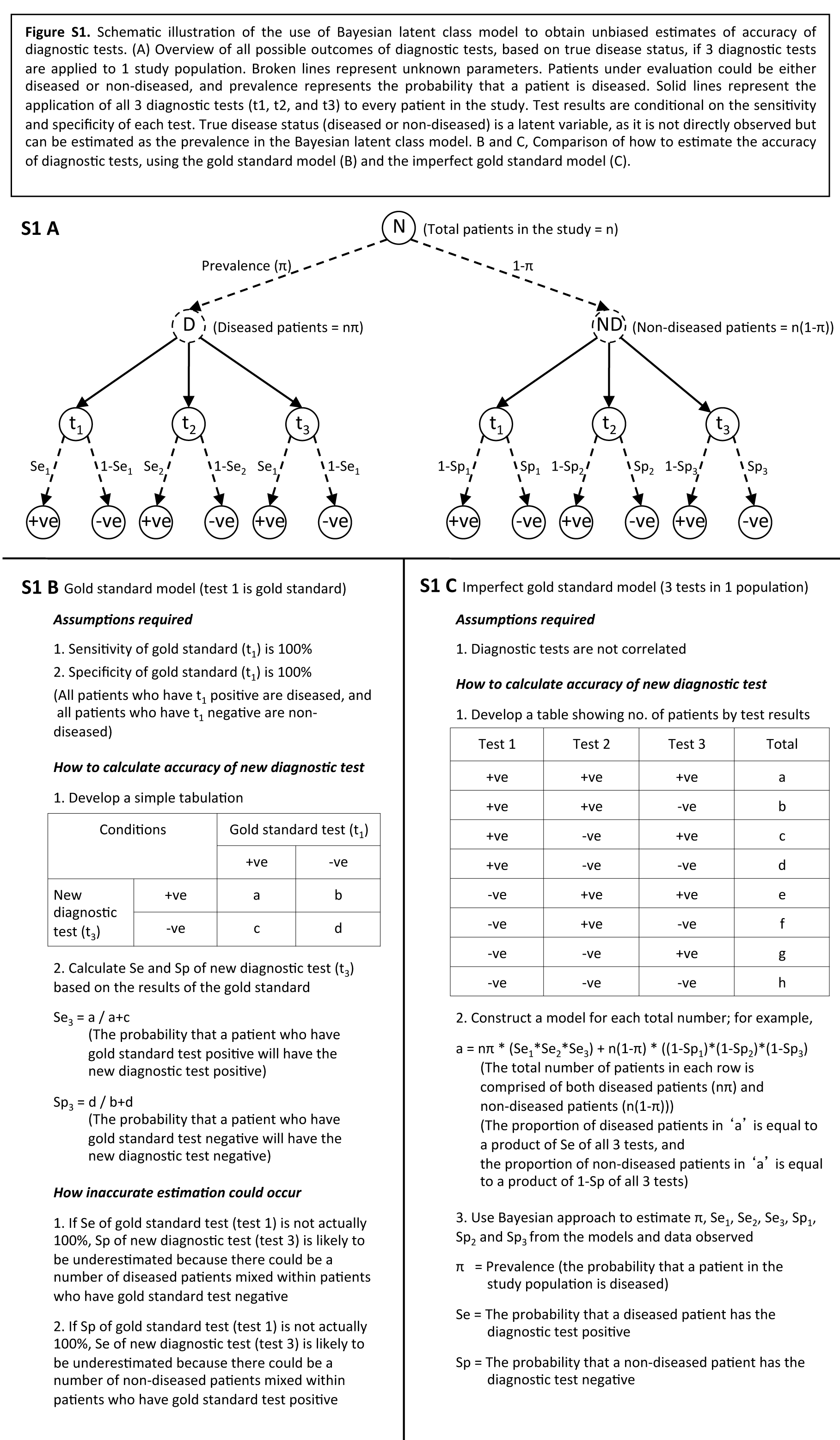

Supplement: Figure S1 — Schematic illustration of the use of Bayesian latent class model (LCM) to obtain unbiased estimates of accuracy of diagnostic tests. (TIF) [file pone.0079489.s001.tif]

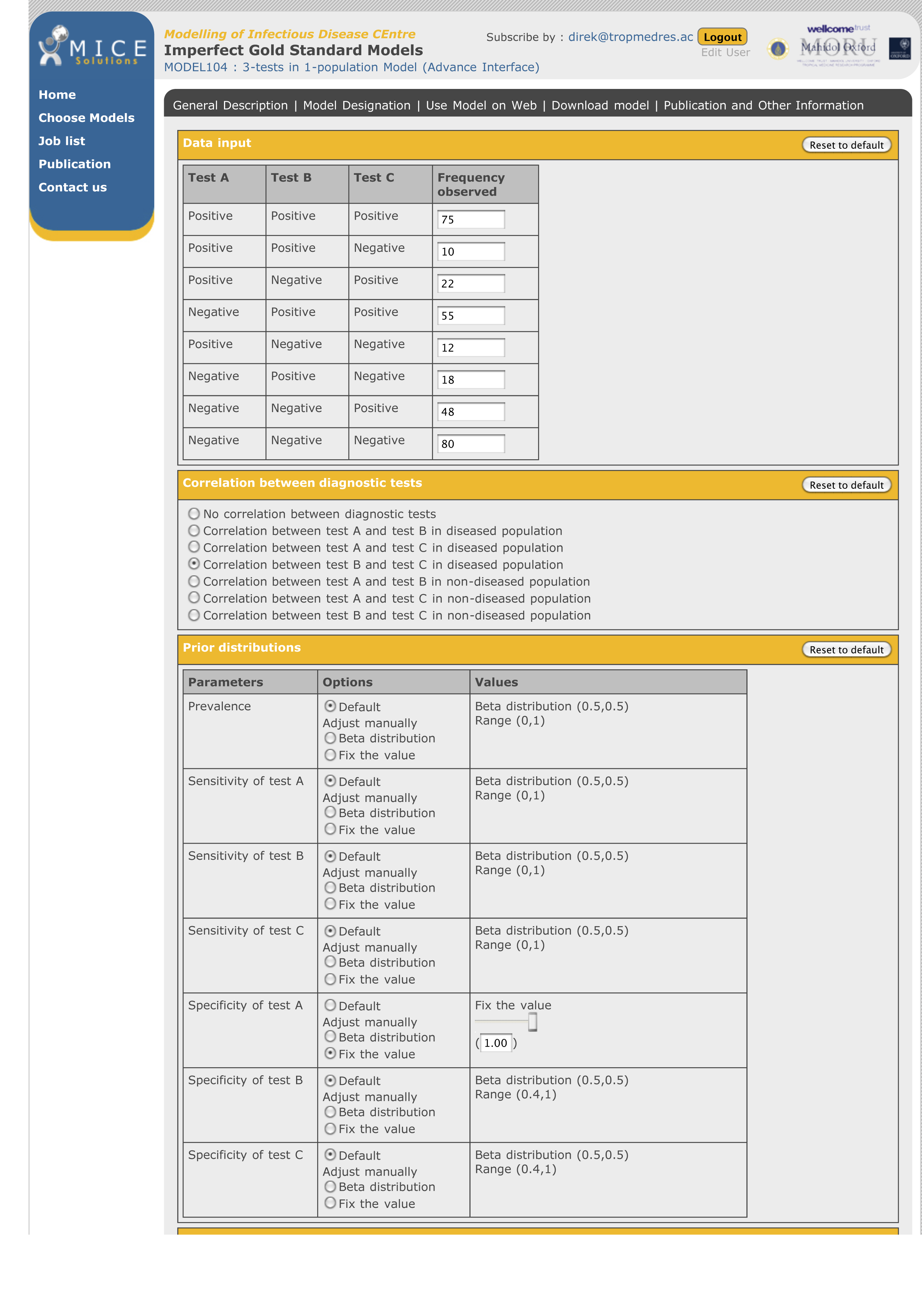

Supplement: Figure S2 — Input screen for the advanced interface of three-tests in one-population model (Walter and Irwig model) provided on the website (http://mice.tropmedres.ac). (TIF) [file pone.0079489.s002.tif]
