# Supplementary material for: Using a Web-Based Application to Define the Accuracy of Diagnostic Tests When the Gold Standard Is Imperfect
Source: PLoS One. 2013 Nov 12;8(11):e79489. doi: 10.1371/journal.pone.0079489 (PMC3827152; doi:10.1371/journal.pone.0079489)
Supplement: Table S1 — Prevalence, sensitivities and specificities for an example data set estimated by the Bayesian latent class model (LCM) using web-based applications and by the formula originally described by Hui and Walter (two-tests in two-population model). (DOCX) [file pone.0079489.s003.docx]

**Table S1.** Prevalence, sensitivities and specificities for an example data set estimated by the Bayesian latent class model (LCM) using web-based application and by the formula originally described by Hui and Walter.

| Parameters | Bayesian LCM  (95% credible interval) | Hui and Walter model  (95% confidence interval) |
| --- | --- | --- |
| Prevalence  Population 1  Population 2 | 2.7 (1.6 to 4.4)  71.6 (69.1 to 74.1) | 2.7 (2.0 to 3.4)  71.7 (70.4 to 73.0) |
| Test A  Sensitivity  Specificity | 96.7 (95.1 to 97.9)  99.3 (98.3 to 99.8) | 96.6 (95.9 to 97.3)  99.3 (98.9 to 99.7) |
| Test B  Sensitivity  Specificity | 96.9 (95.5 to 98.0)  98.4 (97.0 to 99.3) | 96.9 (96.3 to 97.5)  98.4 (97.8 to 99.0) |
