# Supplementary material for: Using a Web-Based Application to Define the Accuracy of Diagnostic Tests When the Gold Standard Is Imperfect
Source: PLoS One. 2013 Nov 12;8(11):e79489. doi: 10.1371/journal.pone.0079489 (PMC3827152; doi:10.1371/journal.pone.0079489)
Supplement: Table S2 — Prevalence, sensitivities and specificities for an example data set estimated by the Bayesian latent class model (LCM) using web-based applications and by the maximum likelihood method described by Walter and Irwig (three-tests in one-population model). (DOCX) [file pone.0079489.s004.docx]

**Table S2.** Prevalence, sensitivities and specificities for an example data estimated by the Bayesian latent class model (LCM) using web-based application and by the maximum likelihood method described by Walter and Irwig.

| Parameters | Bayesian LCM  (95% credible interval) | Walter and Irwig model  (95% confidence interval) |
| --- | --- | --- |
| Prevalence | 5.7 (4.3 to 7.8) | 5.4 (3.1 to 7.7) |
| Test A  Sensitivity  Specificity | 75.0 (59.5 to 87.2)  99.0 (98.2 to 99.8) | 76.5 (65.3 to 87.7)  98.9 (98.5 to 99.3) |
| Test B  Sensitivity  Specificity | 62.6 (48.1 to 75.3)  96.5 (95.5 to 97.4) | 64.4 (47.3 to 81.5)  96.5 (96.0 to 97.0) |
| Test B  Sensitivity  Specificity | 73.6 (58.0 to 85.9)  99.1 (98.3 to 99.9) | 74.9 (63.8 to 86.8)  99.0 (98.7 to 99.3) |
