# Supplementary material for: Using a Web-Based Application to Define the Accuracy of Diagnostic Tests When the Gold Standard Is Imperfect
Source: PLoS One. 2013 Nov 12;8(11):e79489. doi: 10.1371/journal.pone.0079489 (PMC3827152; doi:10.1371/journal.pone.0079489)
Supplement: Table S3 — Settings for the Bayesian latent class models (LCMs) used in the web-based application (http://mice.tropmedres.ac) for two-tests in two-population model (Hui and Walter model). (DOCX) [file pone.0079489.s005.docx]

**Table S3.** Settings for the Bayesian latent class models (LCMs) used in web-based application (<http://mice.tropmedres.ac>) for two-tests in two-population model (Hui and Walter model)

| Settings | Simplified interface (fixed settings) | Advanced interface |
| --- | --- | --- |
| Number of chains ^a^ | 2 | 2 (fixed) |
| Correlation among diagnostic tests | None | None (fixed) |
| Prior distribution | Beta distribution (1,1) for all parameters ^b,c^ | Beta distribution with any two positive numbers ^b,c^ |
| Possible range of all parameters ^c^ | From 0.4 to 1.0 (40% to 100%) for specificities of test A and B ^d^  From 0 to 1.0 (0% to 100%) for the remaining parameters | Adjustable from 0 to 1.0 for all parameters  Can fix any parameter at any value from 0 to 1.0 |
| Initials for Chain 1 | 0.9 for all parameters | Adjustable from 0 to 1.0 for all parameters |
| Initials for Chain 2 | 0.99 for specificities of test A and B  0.7 for the remaining parameters | Adjustable from 0 to 1.0 for all parameters |
| Number of burn-in iterations ^e^ | 5,000 | Adjustable from 1,000 to 200,000 |
| Number of iterations used | 20,000 | Adjustable from 2,000 to 200,000 |
| Number of thinning ^f^ | 1 | Adjustable from 1 to 500 |
| Conventional method to be compared with Bayesian LCMs | Considering test A as gold standard | Adjustable from test A, test B, and combination of both tests as gold standard |

^a^ The number of chains to be simulated in Markov Chain Monte Carlo (MCMC) algorithm

^b^ Beta distribution is a continuous probability density with the interval from 0 and 1.0 (0% to 100%). This distribution is usually used for probability distribution.

^c^ Parameters include (1) prevalence of population 1, (2) prevalence of population 2, (3) sensitivity of test A, (4) specificity of test A, (5) sensitivity of test B and (6) specificity of test B.

^d^ Possible range of specificities of test A and B were set as from 0.4 to 1.0 to avoid the common problem that the model predicts the accuracy of the test the other way around (considering test with true sensitivity of 95% and specificity of 95% as a test with sensitivity of 5% and specificity of 5%).

^e^ For MCMC, a number of iterations prior to the convergence of chains simulated needs to be discarded. The discarded iterations were called burn-in iterations.

^f^ Number of every X iteration to be used for estimating parameters. Setting as 1 means that every iteration will be used.
