# Supplementary material for: Using a Web-Based Application to Define the Accuracy of Diagnostic Tests When the Gold Standard Is Imperfect
Source: PLoS One. 2013 Nov 12;8(11):e79489. doi: 10.1371/journal.pone.0079489 (PMC3827152; doi:10.1371/journal.pone.0079489)
Supplement: Text S1 — Six example data sets of melioidosis suspected patients. (DOCX) [file pone.0079489.s007.docx]

**Text S1.** Six example data sets of melioidosis suspected patients. Observed frequency represents the total number of subjects who had the test result profile in that row.

Example data set 1

| Culture | IHA | IgM ICT | Observed frequency |
| --- | --- | --- | --- |
| positive | positive | positive | 75 |
| positive | positive | negative | 10 |
| positive | negative | positive | 22 |
| negative | positive | positive | 55 |
| positive | negative | negative | 12 |
| negative | positive | negative | 18 |
| negative | negative | positive | 48 |
| negative | negative | negative | 80 |

Example data set 2

| Culture | IHA | IgG ICT | Observed frequency |
| --- | --- | --- | --- |
| positive | positive | positive | 84 |
| positive | positive | negative | 1 |
| positive | negative | positive | 20 |
| negative | positive | positive | 61 |
| positive | negative | negative | 14 |
| negative | positive | negative | 12 |
| negative | negative | positive | 41 |
| negative | negative | negative | 87 |

Example data set 3

| Culture | IHA | ELISA | Observed frequency |
| --- | --- | --- | --- |
| positive | positive | positive | 78 |
| positive | positive | negative | 7 |
| positive | negative | positive | 20 |
| negative | positive | positive | 40 |
| positive | negative | negative | 14 |
| negative | positive | negative | 33 |
| negative | negative | positive | 14 |
| negative | negative | negative | 114 |

Example data set 4

| Culture | IgM ICT | IgG ICT | Observed frequency |
| --- | --- | --- | --- |
| positive | positive | positive | 92 |
| positive | positive | negative | 5 |
| positive | negative | positive | 12 |
| negative | positive | positive | 73 |
| positive | negative | negative | 10 |
| negative | positive | negative | 30 |
| negative | negative | positive | 29 |
| negative | negative | negative | 69 |

Example data set 5

| Culture | IgM ICT | ELISA | Observed frequency |
| --- | --- | --- | --- |
| positive | positive | positive | 83 |
| positive | positive | negative | 14 |
| positive | negative | positive | 15 |
| negative | positive | positive | 40 |
| positive | negative | negative | 7 |
| negative | positive | negative | 63 |
| negative | negative | positive | 14 |
| negative | negative | negative | 84 |

Example data set 6

| Culture | IgG ICT | ELISA | Observed frequency |
| --- | --- | --- | --- |
| positive | positive | positive | 95 |
| positive | positive | negative | 9 |
| positive | negative | positive | 3 |
| negative | positive | positive | 52 |
| positive | negative | negative | 12 |
| negative | positive | negative | 50 |
| negative | negative | positive | 2 |
| negative | negative | negative | 97 |
